# Impact of High Body Weight on Mortality in Critically Ill Patients Receiving Meropenem for Pneumonia

**DOI:** 10.7759/cureus.6403

**Published:** 2019-12-17

**Authors:** Xiaofang Gao, Liling Liang, Peng Yan

**Affiliations:** 1 Department of Pulmonology, Chinese People's Liberation Army General Hospital, Beijing, CHN

**Keywords:** body weight, pneumonia, mortality, meropenem

## Abstract

Introduction: Body size has a significant impact on pharmacokinetics of antibiotics, and leaving body size out of consideration may increase the probability of therapeutic failure. The objective of this study is to evaluate the impact of high body weight on mortality in critically ill patients who received meropenem for pneumonia.

Methods: The electronic medical records of a respiratory intensive care unit (RICU) were retrospectively screened for patients with the first diagnosis of pneumonia and receiving meropenem from 2014 to 2018. Clinical variables and outcome measures were collected in patients who met inclusion criteria. Patients were assigned to quartiles based on body weight (quartile (Q) 1 <52.25 kg; Q2 52.25-65.00 kg; Q3 65.01-72.85; Q4 >72.85 kg). Logistic regression analyses were used to investigate associations between the body weight and mortality. Mortality rates were compared among different body weight quartiles.

Results: Of 1,621 patients admitted to the RICU, 146 patients met the inclusion criteria. There was no significant difference in mortality of patients in different body weight quartiles. In subgroup analysis of patients receiving mechanical ventilation, there was an association between body weight and RICU mortality (odds ratio, 2.97 [1.128-7.846]; P=0.027). In Q4, mortality rate of patients who received meropenem 2 g/day was significantly higher than that of patients who received 3 g/day (69.56% vs 35.71%; P=0.047).

Conclusions: There was an association between body weight and mortality in patients receiving mechanical ventilation. Compared with low-dose meropenem, high-dose meropenem might decrease mortality rate of critically ill patients with highest body weight.

## Introduction

Although there is a wide variability of body size and composition among adults and the tremendous increase in obesity in the past three decades, adult antibiotic recommendations are not based upon body size or composition [1]. In clinical practice, adults who weigh 50 kg are routinely prescribed the same dose of antibiotic as those who weigh 90 kg. Since body size and composition characteristics have a significant impact on pharmacokinetic (PK) of antibiotics and further alters drug serum concentrations, leaving body size out of consideration may potentially increase the probability of therapeutic failure, allow for the emergence of resistant microbial isolates, or lead to unnecessary toxicities [2,3].

The consideration of body size of patients is essential for the optimization of antibiotic therapy in critical care [4]. Meropenem plays a critically important role in controlling severe infections in intensive care unit (ICU) for its broad spectrum activity and low toxicity [5]. However, the fixed dosage of meropenem that is used in real-world situations does not take into account the individual body weight or body size, and it is unknown whether equal dosing of meropenem in patients with widely variable weights is equally efficacious [6]. The aim of this study was to evaluate the impact of high body weight on mortality in patients who received meropenem for severe pneumonia in respiratory ICU (RICU).

## Materials and methods

Study design and patients

This study was conducted by a retrospective chart review of all patients admitted to the RICU of Chinese People's Liberation Army (PLA) General Hospital from August 1, 2014, to December 31, 2018. The RICU has 20 beds and admits a mean of 400 patients with critical respiratory disease every year. Adult patients who had the first diagnosis of pneumonia in electronic medical records and received meropenem for at least three days were included in the analysis. Concomitant antibiotic therapy with suitable indication was allowed. Exclusion criteria were age less than 18 years, pregnancy, lactation, allergy to β-lactam antibiotics, AIDS, neutropenia (white blood cells <1×10^3^/mm^3^), solid or hematologic tumor, and creatinine clearance less than 50 mL/min using the Cockcroft-Gault equation [7]. The study protocol was approved by the Institutional Review Board of Chinese PLA General Hospital.

Data collection

Baseline data obtained from admission information included age, sex, height, weight, APACHE II (Acute Physiology and Chronic Health Evaluation II) score, creatinine clearance, and comorbidities. The comorbid conditions included chronic obstructive pulmonary disease (COPD), diabetes mellitus (DM), hypertension, and coronary artery disease (CAD). The diagnosis of comorbidities was made based on the clinical practice guidelines. Outcomes of interest were all-cause RICU mortality, RICU length of stay (LOS), the need for invasive mechanical ventilation, and the duration of meropenem therapy.

Statistical analysis

Patients were divided into four quartiles according to the distribution of patients’ body weight. Based on admission body weight, the cohort was divided into quartiles of increasing body weight: less than 52.25 kg (quartile[Q]1), 52.25 to 65.00 kg (Q2), 65.01 to 72.85 kg (Q3), and greater than 72.85 kg (Q4). Baseline characteristics were compared among the four groups. Continuous variables were expressed as means with standard deviations. Categorical values were expressed as proportions or percentages. Comparisons were performed using analysis of variance and Kruskal-Wallis test for continuous variables. Categorical variables were compared using χ2 or Fisher exact tests. Logistic regression analyses were used to investigate associations between the body weight and mortality. Mortality rates were compared among different body weight quartiles and predefined subgroup of patients who received different dosage of meropenem. Significance was defined as P value <0.05, and all tests were two-sided. Data were analyzed using the SPSS statistical package (version 19; Chicago, IL, 2008).

## Results

During the four-year period of this retrospective study, 1,621 patients were admitted to RICU for critical pulmonary disease. Among them, 146 patients met the inclusion criteria and were included in the analysis. The average body weight of those patients was 63.22 kg. The dosage of meropenem was determined by attending doctor, either 0.5 g every six hours (2 g/day) or 1 g every eight hours (3 g/day). Table 1 shows the clinical characteristics of each body weight quartile. Among the study population, we found no significant differences in age, gender, severity of illness, concomitant antibiotic therapy or creatinine clearance rate among body weight quartiles. In total, 35.61% of patients were females, with distribution weighted toward the Q1, but there was no significant difference among body weight quartiles. The most frequently used concomitant antibiotics were fluconazole, caspofungin, teicoplanin, and linezolid. There was no marked difference across body weight quartiles for prevalence of COPD, DM, hypertension, and CAD. The results of sputum or secretion culture showed that the pathogens were mainly gram-negative bacteria, accounting for 76.28%. The leading pathogens were Pseudomonas aeruginosa (29.05%) and Acinetobacter baumannii (17.56%).

**Table 1:** Characteristics of the study population by body weight quartiles. APACHE, Acute Physiology and Chronic Health Evaluation; COPD, chronic obstructive pulmonary disease; DM, diabetes mellitus; CAD, coronary artery disease; Cr, creatinine

	Quartile 1 (<52.25 kg) n=37	Quartile 2 (52.25-65.00 kg) n=35	Quartile 3 (65.01-72.85 kg) n=37	Quartile 4 (>72.85 kg) n=37	All n=146	P-value
Age mean (SD)	69.94 (18.06)	61.94 (20.51)	63.83 (16.59)	63.89 (19.88)	64.94 (18.85)	0.29
Female n (%)	17(45.94)	14 (40.00)	10 (27.02)	11 (29.72)	52 (35.61)	0.28
Body weight mean (SD)	45.84 (5.37)	58.34 (3.17)	68.03 ( 2.43)	80.44 (8.05)	63.23 (13.83)	<0.001
Cr mean (SD)	107.88 (39.25)	101.00 (32.61)	104.92 (38.70)	96.55 (38.46)	102.62 (35.31)	0.63
APACHE II mean (SD)	25.26 (7.65)	24.15 (7.45)	26.16 (9.57)	27.14 (8.99)	25.11 (8.71)	0.69
Concomitant antibiotic therapy n (%)	21 (56.75)	20 (57.14)	23 (62.16)	25 (67.56)	89 (60.95)	0.79
COPD n (%)	6 (16.21)	8 (22.85)	5 (13.51)	7 (18.91)	26 (17.81)	0.81
DM n (%)	5 (13.51)	4 (11.42)	3 (8.10)	6 (16.21)	18 (12.32)	0.75
Hypertension n (%)	8 (21.62)	9 (25.71)	8 (21.62)	10 (27.01)	35 (23.97)	0.92
CAD n (%)	8 (21.62)	9 (27.71)	5 (13.51)	11 (29.72)	33 (22.60)	0.38

Outcome data for each body weight quartiles are summarized in Table 2. Mortality in Q4 showed an increasing trend compared with that in other quartiles, but there was no statistical significance. There were no significant differences in the RICU LOS, duration of meropenem therapy, and the proportion of patients who need invasive mechanical ventilation among different body weight quartiles.

**Table 2:** Outcome measures of the study population by body weight quartiles. RICU, respiratory intensive care unit; LOS, length of stay

	Quartile 1 (<52.25 kg) n=37	Quartile 2 (52.25-65.00 kg) n=35	Quartile 3 (65.01-72.85 kg) n=37	Quartile 4 (>72.85 kg) n=37	All n=146	P-value
RICU mortality n (%)	10 (45.94)	9 (48.57)	14 (48.64)	11 (56.75)	73 (50.00)	0.80
RICU LOS mean (SD)	17.78 (10.16)	15.71 (8.13)	17.63 (9.16)	15.56 (7.85)	16.68 (8.85)	0.57
Duration of meropenem therapy mean (SD)	10.10 (5.33)	9.78 (4.63)	11.36 (4.31)	9.62 (3.84)	10.17 (4.56)	0.51
Invasive mechanical ventilation n (%)	15 (40.54)	16 (45.71)	15 (40.54)	12 (32.43)	58 (39.72)	0.63

In logistic regression analysis, the body weight as a continuous variable was not associated with RICU mortality (odds ratio, 0.79 [0.529-1.169]; P=0.23). However, in subgroup analysis of patients who received invasive mechanical ventilation, there was an association between body weight and RICU mortality (odds ratio, 2.97 [1.128-7.846]; P=0.027).

**Figure 1:**
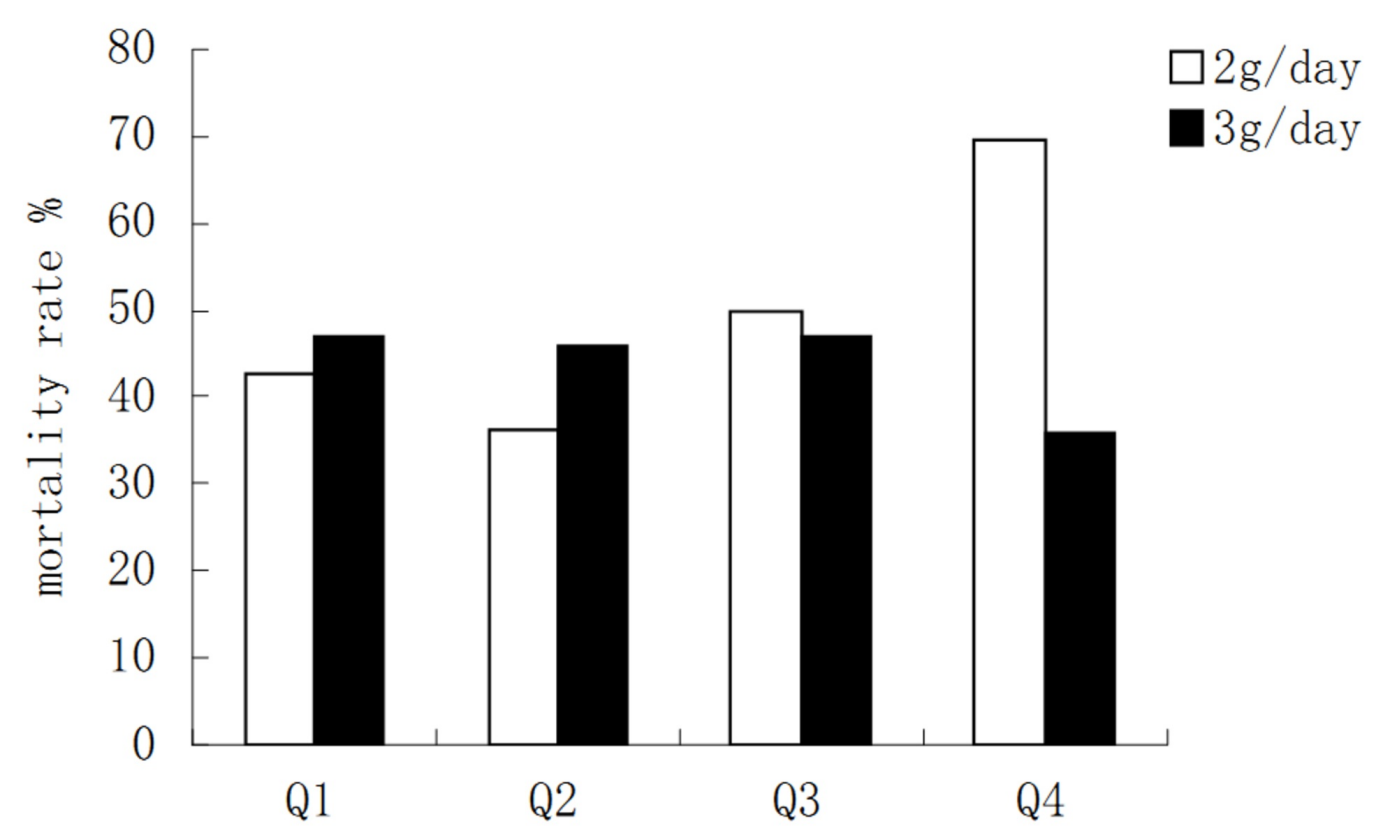
Mortality rate of patients receiving meropenem 2 g/day and 3 g/day in different body weight quartiles. Quartile (Q) 1 <52.25 kg; Q2 52.25-65.00 kg; Q3 65.01-72.85; Q4 >72.85 kg.

In Q4, mortality rate of patients who received meropenem 2 g/day was 69.56% (15/23), which was significantly higher than that of patients who received 3 g/day (35.71%, 5/14; P=0.047). There was no significant difference in mortality rate among other quartiles (Q1, P=0.79; Q2, P=0.59; Q3, P=0.85; Figure 1).

## Discussion

In this retrospective study, we could not detect significant difference in outcome measures among different body weight quartiles, including mortality, RICU LOS, and duration of meropenem therapy. However, in a subgroup of patients who received invasive mechanical ventilation, there was an association between body weight and mortality. Further more, in highest body weight quartile, the mortality rate of patients receiving high-dose meropenem was significantly lower than that of patients receiving low-dose meropenem.

The first antibiotic choice in the treatment of severe infections is carbapenem. Carbapenems are low molecular weight hydrophilic and renally cleared antibiotics. Carbapenems dosage regimens are based on PK data obtained in healthy and nonobese volunteers [8]. However, body size and composition characteristics affect most PK parameters and have a large impact on drug metabolism and distribution [9]. In patients with larger body size, both volume of distribution (Vd) and clearance (Cl) of carbapenems are increased, due to the higher lean body weight, increased plasma volume, and elevated glomerular filtration rate [10]. The increased Vd and Cl can lead to a substantially lower serum concentrations of carbapenems in those patients [11].

In patients with class III obesity, compared with historical control subjects, Vd and Cl of meropenem were increased by 38% and 28%, respectively [12]. Lower serum concentrations of meropenem were observed more often in obese patients than in nonobese patients, and more obese patients had concentrations that did not reach therapeutic targets than nonobese patients [13]. In a study comparing the PK of ertapenem, a significant difference was noted in serum concentration between the high and normal body weight groups, and the area under the concentration curve in the normal weight group was significantly larger than that in high weight patients [14].

When the contribution of PK alterations is unrecognized, patients with high body weight may be underdosed with the use of manufacturer dosing recommendations of carbapenems, and the potential low serum concentrations may induce treatment failure and/or emergence of bacterial resistance [3]. The favorable outcome of critically ill patients with high body weight can be obtained only when serum concentrations reach levels corresponding to PK properties of carbapenems [15]. The association between body weight and mortality found in our study provided evidence for this opinion. Available data support the notion that antibiotics should be given in higher doses to patients with high body weight to better attain pharmacodynamic targets [16]. In patients receiving bariatric surgery, the postoperative infection rates of high and normal weight patients were 16.5% and 2.5%, respectively, at 1 g dosing of perioperative cefazolin. When the dose of cefazolin was doubled, infection rate in the high weight patients dropped to 5.6% [17]. In the present study, we also found high-dose meropenem might decrease mortality rate of critically ill patients with highest body weight.

Many different dosing strategies may be applied to limit the effects of body weight on antibiotic serum concentrations. Tailoring the dosing of antibiotic to the physical characteristics of individual patients could be an important way to achieve maximum effectiveness and safety of antibiotic therapy [18]. For fixed-dose drugs, use of a higher approved dose range in patients with higher body weight may be warranted. However, the interaction between the PK of carbapenems and body weight is complex, and the accuracy of dosing strategies with hydrophilic carbapenems is challenging when treating severe pneumonia in critically ill patients with high body weight [19]. Under such circumstances, the vigilant use of therapeutic drug monitoring may be helpful in these patients [20].

Our study has several limitations that should be taken into account when interpreting the results. Because this is a retrospective study, there are limitations common to such a study design. First, complete data collection is impaired by the absence of any information not in the electronic medical record, and weight data were not collected in the most stringent fashion. Second, concomitant antibiotic therapy reduces the validity of conclusions about the only impact of meropenem. Third, the present study is representative of relatively small group of obese critically ill patients, and further large-scale clinical trials with prospective design should be carried out to confirm our findings.

## Conclusions

There was an association between body weight and mortality in critical ill patients who received invasive mechanical ventilation. Compared with low-dose meropenem, high-dose meropenem might decrease mortality rate of critically ill patients with highest body weight. Although there are several limitations in the present study, the findings have clearly shown that individual body weight could substantially affect clinical effectiveness of meropenem in critically ill patients, and the rationality of one-size-fits-all strategy for prescribing meropenem should be questioned.
